# The dual role of autophagy in acute myeloid leukemia

**DOI:** 10.1186/s13045-022-01262-y

**Published:** 2022-05-07

**Authors:** Wonhyoung Seo, Prashanta Silwal, Ik-Chan Song, Eun-Kyeong Jo

**Affiliations:** 1 Infection Control Convergence Research Center, Chungnam National University College of Medicine, Daejeon, 35015 Korea; 2 Department of Microbiology, Chungnam National University College of Medicine, Daejeon, 35015 Korea; 3 Department of Medical Science, Chungnam National University College of Medicine, Daejeon, 35015 Korea; 4 Division of Hematology/Oncology, Department of Internal Medicine, Chungnam National University College of Medicine, Daejeon, 35015 Korea

**Keywords:** Acute myeloid leukemia, Autophagy, Apoptosis, Therapeutics

## Abstract

Acute myeloid leukemia (AML) is a severe hematologic malignancy prevalent in older patients, and the identification of potential therapeutic targets for AML is problematic. Autophagy is a lysosome-dependent catabolic pathway involved in the tumorigenesis and/or treatment of various cancers. Mounting evidence has suggested that autophagy plays a critical role in the initiation and progression of AML and anticancer responses. In this review, we describe recent updates on the multifaceted functions of autophagy linking to genetic alterations of AML. We also summarize the latest evidence for autophagy-related genes as potential prognostic predictors and drivers of AML tumorigenesis. We then discuss the crosstalk between autophagy and tumor cell metabolism into the impact on both AML progression and anti-leukemic treatment. Moreover, a series of autophagy regulators, i.e., the inhibitors and activators, are described as potential therapeutics for AML. Finally, we describe the translation of autophagy-modulating therapeutics into clinical practice. Autophagy in AML is a double-edged sword, necessitating a deeper understanding of how autophagy influences dual functions in AML tumorigenesis and anti-leukemic responses.

## Background

Acute myeloid leukemia (AML) is a common blood tumor that impairs bone marrow (BM) function. Currently, treatment of AML depends on intensive chemotherapy and/or allogeneic hematopoietic stem cell transplantation. However, many patients are older adults, intolerant to intensive treatment, and highly prone to developing chemoresistant disease secondary to cytotoxic drugs [1–3]. There is an urgent need for more effective and less toxic therapeutics for AML [1, 4]. Autophagy is an intracellular catabolic pathway by which cytoplasmic contents, including damaged organelles and protein aggregates, are sequestered into autophagosomes for lysosomal degradation [5]. Autophagy is linked to the pathogenesis of several major human diseases, including cancers [5–7]. The autophagy-related signature helps predict the overall survival (OS) and/or clinical outcomes of patients with AML. In addition, autophagy may be involved in AML initiation, progression, and response to chemotherapy [8, 9], thereby shedding light on autophagy-modulating therapeutics.

The role of autophagy in tumorigenesis and the anticancer response of AML are unclear. Earlier studies have implicated autophagy in AML survival and tumorigenesis [10], supported by findings that autophagy-inducing catalytic mTOR inhibitors promote leukemic cell survival [11]. In addition, autophagy induction is linked to a prosurvival response during chemotherapy to suppress treatment efficiency [12]. By contrast, loss of autophagy in hematopoietic stem cells results in expansion of a progenitor population, principally invasive myeloproliferative cells in BM, in human AML [13]. Moreover, autophagy promotes leukemic cell death via degradation of fusion oncoproteins, including promyelocytic leukemia (PML)–retinoic acid receptor α (RARA) and breakpoint cluster region–Abelson kinase (BCR–ABL) [14–17]. Thus, a better understanding of the roles of autophagy in AML tumor cells and the tumor microenvironment would enable the development of novel AML treatments and improve the outcomes of patients harboring specific mutations.

Here, we first discuss the overview of AML and autophagy in autophagy regulation in hematopoiesis and leukemogenesis. We then divided our review into three sections. In the first part, we discuss the multifaceted functions of autophagy in the context of various genetic alterations of AML. In the second part, we describe the recent progress on the autophagy-related genes (ATGs) as potential biomarkers in AML. We also discuss the recent findings that provided insight into the crosstalk among autophagy, mitochondria, and metabolism. Finally, we describe autophagy-modulating pathways (activating, inhibitory, or combined action with apoptosis) to develop novel therapeutics for AML. Emphasis is placed on autophagy-targeting agents, which have shown potential promise in clinical trials. These efforts may help to facilitate the autophagy-based development of diagnosis, prediction of prognosis, or targeted treatments for patients with AML.

### Overview of AML

#### AML classification

AML is a severe hematologic malignancy caused by transformation and proliferation of myeloid progenitors in the presence of BM failure. AML is the most common form of acute leukemia in adults and is prevalent in older patients (median age at diagnosis in the USA: 67 years). It also has heterogeneous and poor prognostic features, including secondary AML and treatment-related AML [18, 19]. The pathogenesis of AML is related to environmental stresses (*e*.*g*., ionizing radiation, chemical exposure, and retroviral infection) and genetic alterations associated with leukemogenesis.

AML can be diagnosed based on the blast percentage and cytogenetic abnormalities. In the WHO classification, the threshold for diagnosing AML is ≥ 20% blasts in BM or peripheral blood. In addition, patients who have defective hematopoiesis with specific clonal cytogenetic abnormalities (including t(15;17), t(8;21), inv(16), and t(16;16)) are diagnosed with AML irrespective of the blast percentage. According to morphology, immunophenotype, genetics, and clinical characteristics, AML is categorized into six groups [3, 20]. Although the molecular AML classification has been updated, the mainstay of treatment has not changed in > 40 years [21, 22]. The standard induction therapy protocol for AML, excluding acute promyelocytic leukemia (APL), is Ara-C with anthracycline in young adults (< 60 years). In older adults (> 75 years) or ineligible patients (those unable to tolerate the toxicity of standard chemotherapy), low-dose Ara-C or low-intensive chemotherapy (5-azacytidine, decitabine) are alternatives. For this reason, older patients with AML exhibit particularly poor outcomes, with less than 5% of patients surviving five years after diagnosis, compared with 40% of young patients [23, 24]. Therefore, novel, innovative therapeutic approaches to improve the CR rate or increase the duration of remission are needed.

#### The genetic alterations of AML

The genetic alterations of AML are complicated and generally categorized into nine groups: signaling molecules (59%), DNA methylation (44%), chromatin modification (30%), nucleophosmin (*NPM1*) (27%), myeloid transcription factor (22%), transcription factor fusion (18%), tumor suppressor (16%), spliceosome complex (14%) and cohesion complex (13%) [25]. Several such genetic alterations in hematopoietic progenitors induce leukemogenesis and distort normal hematopoiesis, resulting in uncontrollable expansion of blasts and BM failure. For example, *NPM1* mutation, which is harbored by one-third of patients with AML, may be associated with enhanced self-renewal of hematopoietic progenitors and may promote AML leukemogenesis [26, 27]. FMS-like tyrosine kinase 3 (*FLT3*) mutation occurs in about 30% of all AML cases [28]. *FLT3* is a class III tyrosine kinase receptor gene; if it has internal tandem duplication (ITD) or mutation in the tyrosine kinase domain, it can cause a proliferation advantage for hematopoietic progenitor cells. *FLT3*-ITD is a driver mutation that presents with a high leukemic burden and confers a poor prognosis in patients with AML. Recently, several targeted agents for *FLT3* mutation, such as midostaurin and gilteritinib, have demonstrated their effectiveness for clinical outcomes in AML patients [29, 30]. When *NPM1* mutation is combined with *FLT3*-ITD mutation in knock-in mouse models, it is observed to show more AML phenotypes, suggesting a potent molecular synergy between the two mutations [31].

AML with t(8;21)(q22;q22);*RUNX1/RUNX1T1* and inv(16)/t(16;16);*CBFB/MYH11* gene rearrangement, which is called core-binding factor AML (CBF-AML), makes fusion oncoprotein, which leads to transcriptional deregulation and impaired hematopoietic cell differentiation. CBF-AML occurs in approximately 20% of patients with de novo AML. Clinically, both inv(16)/t(16;16) and t(8;21)(q22;q22) AML show high rates of complete remission (CR) and prolonged CR duration, especially following consolidation chemotherapy with high-dose cytarabine (Ara-C), and are thought to have a better prognosis [32]. However, *c-KIT, RAS,* or *FLT3* mutations have a negative impact on the outcome of CBF-AML [33, 34]. Another good example of differentiation arrest is AML with *PML*/*RARA* gene rearrangement. The fusion oncoprotein made by reciprocal translocation of chromosomes 15 and 17 blocks the gene transcription and eventually causes maturation arrest at the promyelocyte stage. Several well-known agents, such as all-trans retinoic acid (ATRA) and arsenic trioxide (ATO), degrade this fusion oncoprotein and facilitate the cell differentiation [35, 36].

AML with isocitrate dehydrogenase 1 and 2 (*IDH1/2*) mutation occurs in about 10% of AML patients. IDH is an enzyme that catalyzes the oxidative decarboxylation of isocitrate, producing alpha-ketoglutarate. Somatic mutation at crucial arginine residues in IDH1 (which is cytoplasmic) and IDH2 (which is mitochondrial) causes the gain of novel enzymatic activity and converts alpha-ketoglutarate to 2-hydroxyglutarate with consumption of nicotinamide adenine dinucleotide phosphate. 2-Hydroxyglutarate supports tumorigenesis by increasing histone and DNA methylation and impairing cell differentiation [37, 38]. Ivosidenib and enasidenib inhibit IDH1 and IDH2, respectively, and are approved by the US Food and Drug Administration for relapsed and refractory AML with those specific mutations. Under enasidenib or ivosidenib treatment, myeloid differentiation and trilineage hematopoietic cell recovery has been observed, so IDH1/2 inhibitors act as differentiation-inducing agents [39, 40]. In addition, mutations in U2 small nuclear RNA auxiliary factor 1 (*U2AF1*) or *Wilms’* tumor suppressor gene 1 (*WT1*) are associated with poor survival of patients with AML and myelodysplastic syndromes [41–43]. As in other malignancies, how the underlying somatic mutations of AML occur and their links to AML pathogenesis are unclear.

### Overview of autophagy and autophagy-related genes

#### Overview of several types of autophagy in AML

Autophagy is an intracellular catabolic process that can eliminate unnecessary or dysfunctional components in lysosomes during stress conditions [44–46]. In the context of cancers, various stresses—including hypoxia, metabolic stresses, genomic alterations, anticancer agents, and radiotherapy—can induce autophagy [8, 9]. Given its fundamental role in organelle quality control and homeostasis, autophagy suppresses the initiation of tumorigenesis. However, autophagy promotes tumor progression by releasing metabolic precursors for ATP generation by tumor cells, particularly in response to therapy-induced stresses [47, 48]. In addition, autophagy plays a multifaceted role in regulating the immune escape of cancer cells [49]. A deeper understanding of autophagy function in tumor cells and the tumor microenvironment will enable the development of novel therapeutic strategies for cancer [50].

Autophagy is classified into macroautophagy, microautophagy, and chaperone-mediated autophagy [51, 52]. The most widely studied of these is macroautophagy, initiated by the formation of phagophores, followed by the generation of double-membrane autophagosomes, which fuse to lysosomes, leading to cargo degradation [47, 53]. Autophagy is tightly orchestrated by a series of ATGs. ATGs show promise as biomarkers and therapeutic targets in cancers, and their expression is epigenetically controlled by microRNAs (miRNAs) and long non-coding RNAs (lncRNAs) [54–56]. There is a relationship between genetic alterations in ATGs and AML risk [57], although the underlying mechanism is unknown.

Autophagy activation is regulated by autophagy signaling pathways, including the pathway centered on mechanistic target of rapamycin (mTOR), a master regulator of energy metabolism for cell growth and biomass expansion [58–60]. The elongation of autophagosomal membranes is mediated by two ubiquitin-like conjugation pathways, the autophagy-related protein (ATG) 8 and ATG12/ATG5/ATG16L1 protein complexes, resulting in the conjugation of phosphatidylethanolamine to mammalian LC3 and conversion of LC3-I into LC3-II, a marker used to monitor autophagy [61, 62]. After fusion to lysosomes, the degraded cytoplasmic cargo can be used for cell survival by recycling precursors such as amino acids [52, 63]. Given their role in nutrient signaling, immune function, and energy metabolism, lysosomes are an important potential target for AML and other cancers [64].

Much progress has been made in identifying selective autophagy receptors, which are responsible for a highly selective mechanism for intracellular quality control involving selective degradation of sizable and dysfunctional cytosolic components [47]. Targeting selective cargo receptors such as p62/sequestosome 1 (SQSTM1) and BNIP3L/Nix has anti-leukemic potential and potential as prognostic markers for AML [8, 65, 66]. However, the role of selective autophagy and its receptor(s) in AML tumorigenesis is unclear. Future studies should evaluate the functions of the various types of autophagy in promoting and suppressing leukemogenesis. The physiological and pathological implications of autophagy on the development of the hematopoietic system and AML tumorigenesis are discussed in the following.

#### Autophagy functions in hematopoiesis and leukemogenesis

Various types of autophagy participate in normal hematopoiesis and AML development [9, 67]. Detailed illustrations of the autophagy function in distinct stages of AML progression are provided elsewhere [8, 9]. This section briefly discusses the several recent findings that shed light on the implications of autophagy functions in hematopoiesis, myeloid cell differentiation, and leukemogenesis. The normal hematopoietic system is maintained by autophagy, as shown by the finding that ATG7 is essential in protecting hematopoietic stem cells (HSCs) from malignant proliferation, accumulation of damaged mitochondria, and DNA damage [13, 67]. Ending autophagy activity in HSCs by the conditional deletion of ATG7 leads to invasive myeloproliferation and increases its lethality in mice [67]. Among the most studied pathways regulating HSC homeostasis, the Akt-mTOR network is critical for self-renewal, survival, and differentiation and prevents the transformation of HSCs into leukemia stem cells [68]. Considering that inhibition of the Akt-mTOR pathway is a well-established strategy for inducing autophagy activation [69], the precise positioning of autophagy in normal hematopoiesis may depend on the proper regulation of autophagy in HSCs.

Besides this fundamental role in maintaining normal hematopoiesis, several lines of evidence suggest that macroautophagy is involved in the regulation of leukemic cell survival and the initiation and progression of AML [8, 9, 70]. Indeed, the inactivation of autophagy genes ATG7 or ATG5 results in the increased survival and suppression of functional LICs, the principal cells in the development and relapse of AML, in a murine AML model [70]. Defective autophagy in mouse models upregulates the NOTCH signaling pathway, which may trigger the initiation of leukemic HSC. AML patients also show low autophagy levels and upregulated NOTCH signaling in hematopoietic stem cells [71]. In addition, the oncogenic mutations of the splicing factor U2AF35 in MDS result in the abnormal processing of ATG7 pre-mRNA and decreasing ATG7 level, thereby contributing to the progression of AML [72]. Also, autophagy and metabolism show crosstalk and cooperation for physiological functions in the hematopoietic system through the maintenance and differentiation of HSCs [73, 74]. For example, an in vivo model with heterozygous loss of Atg5 exhibits a glycolytic shift and aggressive leukemia [75], suggesting autophagic control of glycolysis in leukemic transformation. The detailed crosstalks between autophagy and metabolism will be discussed in the later part of this review.

Several findings show that autophagy plays an essential role in hematopoietic differentiation. For instance, key ATGs involved in various stages of autophagosome formation are significantly downregulated in primary AML compared with healthy granulocytes [76]. The differentiation of granulocytes from AML/APL cells and CD34 + progenitor cells is correlated with upregulated expression of essential autophagy genes such as *ATG3*, *ATG4D*, and *ATG5* [76]. As discussed in the following section, the interaction between PML/RARA and FYVE-domain-containing protein (ALFY/WDFY3), a PI3P-binding autophagy scaffold protein, was found to promote autophagy-mediated proteolysis of PML/RARA and neutrophil differentiation of AML cells [77]. These findings suggest that the increased ATG expression and autophagic activity are related to neutrophil differentiation. Together, the basal level of autophagy and autophagy gene expression is crucial for the physiological development of hematopoiesis, whereas dysregulated autophagy, either up- or downregulated, may contribute to leukemic initiation and progression. Additionally, the effects on myeloid differentiation of autophagy-targeting therapeutics should be considered.

### Genetic alterations and autophagy in AML

**Fig. 1:**
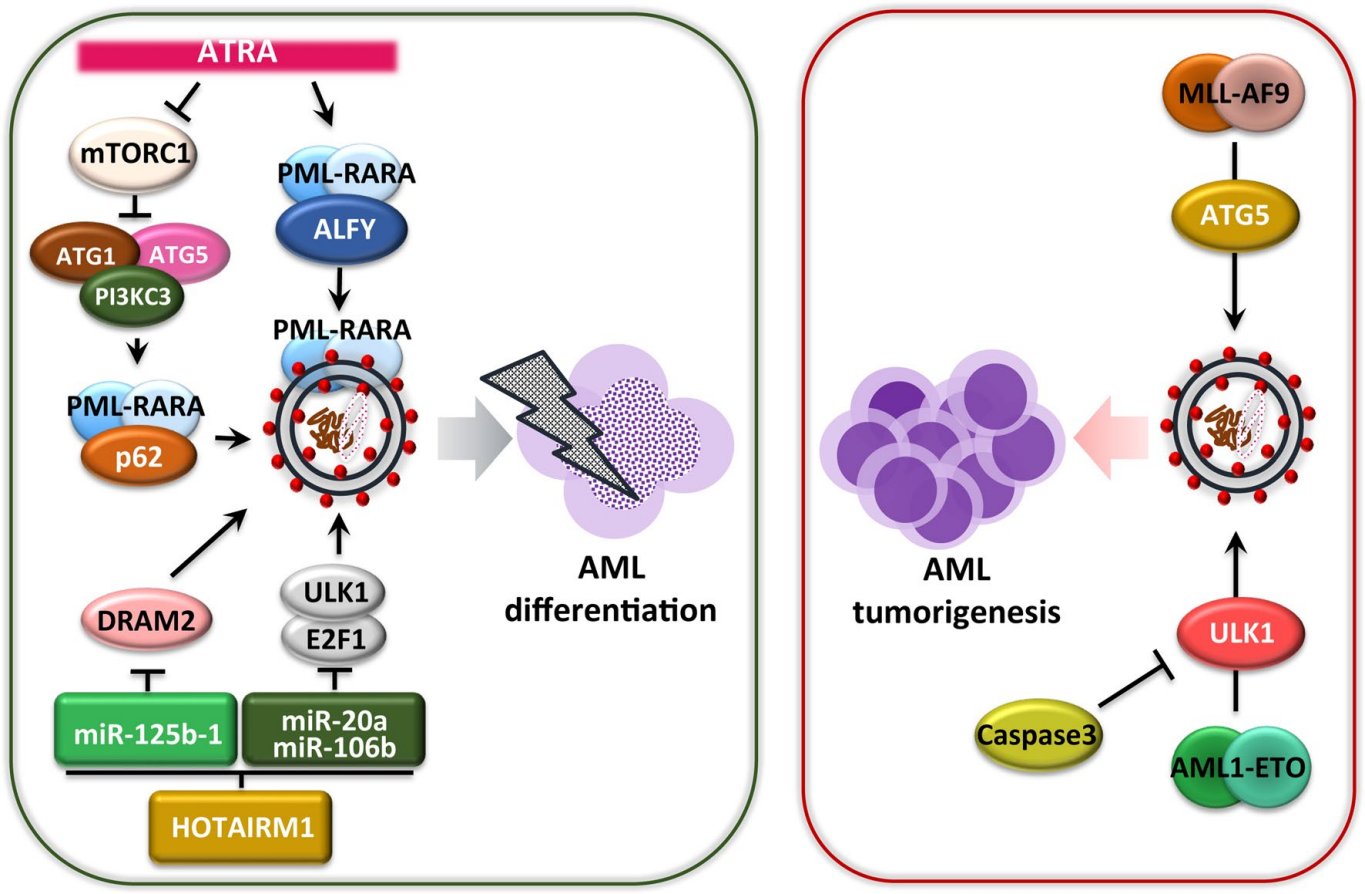
AML tumorigenesis-induced fusion gene linking autophagy. ATRA increases ALFY protein-mediated autophagic degradation of the PML/RARA protein. ATRA inhibits the mTOR1 signaling pathway and then induces p62-mediated autophagic degradation of PML/RARA. The long non-coding RNA HOTAIRM1 acts as a sponge for the microRNAs, miR-20a/106b and miR-125b, targeting the genes *ULK1, E2F1,* and *DRAM2*, which are required for PML/RARA autophagic degradation. In AML with *MLL/AF9* translocation, ATG5 protein-dependent autophagy aggravates the disease status. Another fusion protein, AML1-ETO, functions as an oncoprotein through ULK1-mediated autophagy; the enzyme caspase-3 destroys ULK1, inhibiting the autophagy process and tumorigenesis of AML1-ETO

**Fig. 2:**
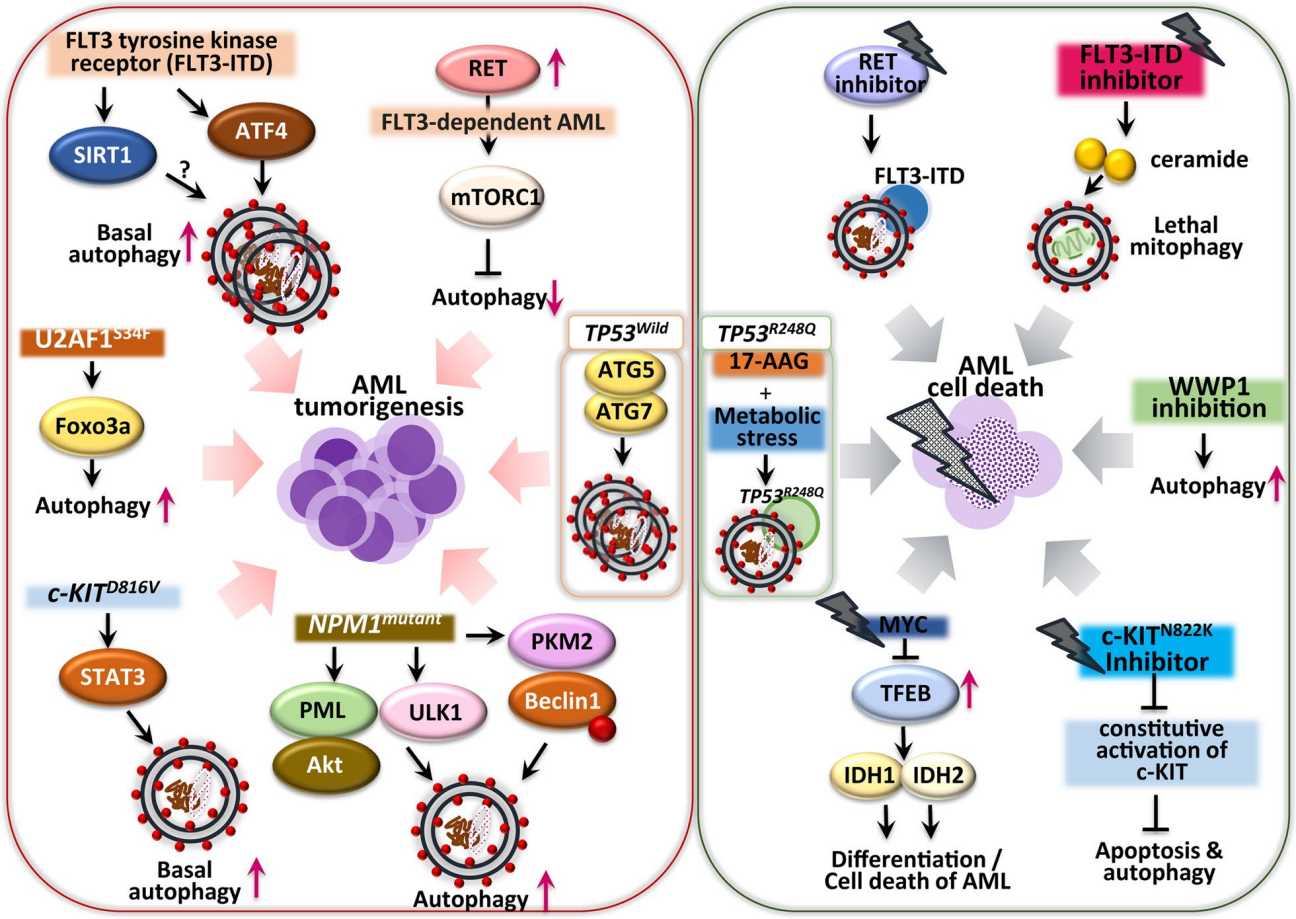
Oncogenic mutations linking autophagy to the tumorigenesis and anticancer effects in AML. The *FLT*-ITD3 mutation in AML cells increases ATF4 protein-mediated basal autophagy to promote tumorigenesis. Overexpression of the enzyme sirtuin 1 is frequently observed in AML patients with *FLT3*-ITD mutation, although the role of sirtuin 1 in AML autophagy is unclear. A RET protein in *FLT3*-dependent AML inhibits autophagy via mTORC1 pathways, while the RET inhibitor suppresses leukemic cell proliferation by autophagic degradation of mutant FLT3. Also, a FLT3-ITD inhibitor induces expression of the lipid ceramide to execute lethal mitophagy. Inactivation of WWP1 induces autophagy to impair tumor growth. Inhibition of c-KIT^N822K^ blocks constitutive activation of c-KIT and induces both apoptosis and autophagy. The transcription factor TFEB induces AML differentiation and apoptosis through the IDH1/2–TET2 axis in Myc protein-deficient conditions. The mutant *NPM1* gene activates leukemogenic autophagy through PML/AKT signaling, ULK1 protein stabilization, and PKM2 enzyme-mediated phosphorylation of the Beclin-1 protein. The *KIT*^*D816V*^ mutation in AML constitutively activates the STAT3 signaling pathway, enhancing basal autophagy to promote AML cell proliferation. The *U2AF1*^*S34F*^ mutation increases autophagy flux through the Foxo3a transcription factor. AML cells with the *TP53*^wild type^ gene can survive through ATG5/7 protein-mediated autophagy. The Hsp70 inhibitor, 17-AAG, under a condition of metabolic stress, induces degradation of *TP53*^*R248Q*^ in AML through chaperone-mediated autophagy

As described in the overview section, chromosomal translocations resulting in gene fusions and oncogenic mutations are found frequently in AML. The oncogenic fusion proteins and genetic mutations associated with autophagy in the context of AML tumorigenesis, and their anticancer effects, have been reviewed extensively in recent studies [8, 9]. In this section, we summarize and update the recent progress that has emphasized the links of autophagy with fusion genes and oncogenic mutations in AML (Figs. 1 and 2).

#### Fusion genes and autophagy in AML

Several AML subgroups are defined by fusion genes generated from chromosome translocations, and the resulting fusion proteins are involved in driving leukemogenesis [9, 10, 78, 79]. Evidence, which continues to accumulate, has highlighted that the modulation of autophagy targeting specific fusion oncoproteins might provide therapeutically beneficial effects for a certain subgroup of AML patients with chromosomal rearrangement [10].

APL constitutes a subgroup of AML (AML-M3) associated with the balanced reciprocal chromosome rearrangement of t(15;17)(q24;q21) that generates the fusion of *PML* and the *RARA* genes [80, 81]. Both ATRA and ATO are effective agents for curing t(15;17) chromosomally translocated APL [82]. Early studies have shown that autophagy activation by mTOR inhibition is critical in the therapy-induced degradation of PML/RARA oncoprotein and differentiation of APL cells [16, 17]. Mechanistically, the oncoprotein PML/RARA interacts with the receptor p62/SQSTM1 that increases the lysosomal delivery and degradation of PML/RARA in human myeloid cells in response to ATRA [16]. Another study showed that ATRA-induced autophagy in AML and APL cells involves an association between PML/RARA and ALFY/WDFY3 [77, 83]. Also, mTOR-dependent autophagy activation is required for ATO-induced NETosis in APL cells [84]. Importantly, the mTOR pathway inhibitor rapamycin shows a synergistic effect with ATO in the induction of NETosis-mediated elimination of leukemia-initiating cells (LICs) in APL cells and in an APL model in vivo [84].

Several epigenetic mechanisms have been reported in terms of autophagy-mediated PML/RARA degradation in APL. For example, increased miRNA has-mir-125b-1 (MIR125B1) suppresses PML/RARA proteolysis through the autophagy pathway by targeting the autophagy gene DNA-damage regulated autophagy modulator 2 (DRAM2) expression in APL cells and in vivo [85]. Chen et al. also showed that the lncRNA HOTAIRM1 is required for ATRA-mediated PML/RARA degradation and differentiation of promyelocytic cells through the induction of autophagy via regulating a pathway involving the microRNAs, miR-20a/106b and miR-125b, and their ATG targets [86]. These observations add to the emerging evidence that autophagy activation should be considered a critical therapeutic target of the PML/RARA oncoprotein and that it reduces leukemic burden, when combined with treatment by ATRA or ATO.

Mixed lineage leukemia (*MLL*) gene translocations associated with poor prognosis are observed in approximately 80% of pediatric AML. The aberrant fusion proteins from MLL support leukemogenesis in the chromosome 11q23-rearranged subgroup in childhood [87]. A previous study showed that the MLL fusion protein MLL-AF6 augments rat sarcoma virus (*RAS*) oncogene activity and is thus targeted by tipifarnib, a RAS inhibitor [88]. Interestingly, chemical suppression of RAS signaling leads to the effective inhibition of t(6;11)-rearranged AML tumors through induction of both apoptosis and autophagy [88]. Another study showed that ATG5 contributes to the development of MLL-AF9-driven leukemia, but not in chemotherapeutically sensitive mice with AML expressing MLL-AF9 [89]. In addition, CBF-AML has chromosomal aberrations with translocation t(8;21) and inversion inv(16), resulting in the production of the AML1-eight-twenty one (ETO) and CBFbeta-MYH11 fusion oncoproteins, respectively [90]. Unc-51-like kinase 1 (ULK1)-mediated autophagy activation can control and delay the AML1-ETO9a-driven leukemogenesis in a caspase-3 knockout mouse model of AML [91], suggesting that crosstalk between apoptosis and autophagy determines the pace of AML1-ETO-driven leukemogenesis. These findings suggest that autophagy or ATGs play a differential role in the initiation, progression, and therapeutic responses in AML cells depending on the distinct type of fusion oncoprotein.

#### *FLT3* mutations linking autophagy in AML

Common mutations in *FLT3* in AML are an internal tandem duplication (*FLT3*-ITD) and a point mutation in the tyrosine kinase domain (*FLT3*-TKD). Both mutations enhance FLT3 kinase activity and the downstream signaling pathway, explaining the aggressive phenotype and poor prognosis of AML [92]. In AML cells, FLT3-ITD expression leads to increased basal autophagy, which is required for AML cell survival, in an ATF4-dependent manner. Inhibition of the transcription factor ATF4 also suppresses autophagy-dependent AML cell growth and reverses overall mouse survival [93], suggesting that targeting the ATF4-dependent autophagy pathway has therapeutic potential for patients with AML and *FLT3* mutations. In addition, inhibition of FLT3-ITD upregulates the synthesis of ceramide, a pro-cell death lipid, to induce lethal executive mitophagy and AML cell death [94]. By contrast, autophagy activation via RET inhibition may contribute to anticancer effects in AML cells via mutant FLT3 degradation. The *RET* gene, which encodes a receptor tyrosine kinase frequently overexpressed in AML, is associated with AML tumorigenesis via mTORC1 signaling and autophagy suppression [95]. RET inhibition results in growth suppression of oncogenic *FLT3*-dependent AML cells, accompanied by upregulation of autophagy. These data suggest a potential strategy for anti-leukemic responses by targeting RET–mTORC1 signaling via autophagy induction [95]. Sirtuin 1, a mammalian nicotinamide adenine dinucleotide-dependent histone deacetylase, is frequently overexpressed in leukemia stem cells from patients with AML and the *FLT3*-ITD mutation [96]. Given the role of sirtuin 1 in autophagy in various physiologic and pathologic conditions [97, 98], further studies should clarify the function of sirtuin 1 in autophagy regulation during AML tumorigenesis.

#### *KIT* mutations and autophagy

*KIT* mutations are associated with higher tumor cell proliferation and a greater risk of AML relapse [33, 99]. Thus, blockade of KIT tyrosine kinase is beneficial for treating KIT-positive tumors, and it has been evaluated in two clinical trials [100–102]. Mechanistically, the *KIT*^D816V^ mutation in AML cells upregulates basal autophagy, promoting AML cell proliferation and survival via STAT3 signaling [103]. These data highlight the potential of targeting STAT3-mediated autophagy to treat AML patients with the *KIT*^D816V^ mutation [103]. Another mutation in c-KIT (N822K T > A) that constitutively activates c-KIT renders AML cells highly sensitive to sunitinib (a tyrosine kinase inhibitor). The increased sensitivity of AML cells to sunitinib results in AML cell death by inducing apoptosis and autophagy pathways [104]. The distinct autophagic features of each KIT receptor tyrosine kinase mutation in AML may contribute to the design of personalized therapeutic approaches for AML.

#### *NPM1* mutations and autophagy

The cytosolic *NPM1* mutant is one of the most frequent genetic alterations in AML. In *NPM1*-mutated AML, autophagic activity is increased, leading to leukemic cell survival [105, 106]. Mechanistically, mutant *NPM1* (*NPM1* mutation type A) interaction with the tumor suppressor PML results in cytoplasmic delocalization and stabilization of PML, thereby promoting autophagy and AML cell proliferation, at least in part by activating AKT signaling [106]. The mutant NPM1 interacts with a core autophagy protein, ULK1, and promotes TRAF6-dependent ULK1 ubiquitination via miR-146 to promote protein stability and autophagic cell survival [107]. In addition, the mutated *NPM1*-induced increase in autophagic activity is mediated by pyruvate kinase isoenzyme M2 (PKM2), a key glycolytic enzyme [105]. PKM2, which is upregulated in *NPM1*-mutated AML and is associated with a poor prognosis, promotes autophagy and Beclin-1 phosphorylation, thereby contributing to tumor cell survival [105]. However, the mechanism by which PKM2-mediated metabolic alteration affects autophagic activities and AML pathogenesis is unclear.

#### *MYC*-driven oncogenesis and the autophagy regulator transcription factor EB (*TFEB*)

The *MYC* oncogene is involved in the tumorigenesis of pediatric and adult AML [108–110]. An autophagy-related mechanism has been proposed for *MYC*-driven oncogenesis of AML. *MYC*-induced AML progression is mediated by transcriptional repression of TFEB, a global regulator of autophagy and lysosome biogenesis [111]. In AML cells, TFEB functions as a tumor suppressor by inducing differentiation and death. Interestingly, TFEB epigenetically induces the expression of *IDH1* and *IDH2*, resulting in global hydroxylation of 5-methylcytosine and induction of critical genes driving monocytic and granulocytic differentiation and apoptosis [111]. Therefore, *TFEB*, originally reported as an oncogene, functions as a tumor suppressor, particularly in MYC family oncoprotein-driven AML.

#### *TP53* and autophagy

Accumulating evidence shows that *TP53* mutations, particularly those giving gain-of-function to p53 proteins, exhibit oncogenic responses and aggressive tumor profiles in a variety of cancers [112]. Compared with solid tumors, *TP53* gene mutations are relatively rare at the diagnosis stage in most hematological cancers, including AML [113, 114]. However, a high incidence of *TP53* mutations features in patients with therapy-related AML and myelodysplastic syndrome [115–117]. *TP53* mutation in therapy-related myeloid neoplasm is associated with increased chromosomal instabilities and genome abnormalities with complex karyotypes [116]. Also, *TP53* mutations correlate with an aggressive clinical outcome and poor prognosis in AML [115, 116, 118]. These findings suggest that p53 dysfunction caused by *TP53* mutations leads to tumor progression and impaired outcomes for AML. However, it remains largely unknown why the p53 mutant variants display pathological inconsistencies and have different clinical consequences in AML. More studies focused on the molecular mechanisms underlying the functional effects of p53 mutants are needed to develop effective personalized therapeutic strategies based on the distinct types of *TP53* mutation.

In the context of autophagy, a previous study showed that autophagy inhibition by hydroxychloroquine and knockdown of ATG5 or ATG7 decreased tumor cell survival in AML harboring wild-type *TP53*, but not in mutated *TP53* [119]. Mechanistically, blockade of autophagy enhances the expression of p53 and activates BAX- and PUMA-dependent apoptotic responses in AML [119], suggesting that intact p53 function is required for autophagy-targeting therapeutics to be effective against AML. This is a limitation with regard to earlier efforts to generalize autophagy-targeting therapeutics in the p53 wild-type myeloid cancers. Despite this, there is evidence that autophagy inhibition may lead to increased apoptosis sensitization of tumor cells through a transcriptional mechanism involving FOXO3a [120]. Notably, a recent study shed light on the role of autophagy in AML cells in the context of p53-mediated apoptosis, which is related to the miR-10a inhibition-induced synergistic cytotoxicity between MDM2 inhibitors and Ara-C [121]. Given that targeting the interaction between p53 and MDM2/MDMX through small-molecule inhibitors has not been a widely tested strategy for overcoming tumorigenesis of p53 wild-type tumors [122], exploring the anti-leukemic efficacy of potential combinatorial strategies between autophagy modulators and MDM inhibitors for the treatment of AML harboring wild-type p53 protein presents a challenge.

For p53 mutant-type AMLs, autophagy-modulating therapeutic strategies may help promote the degradation of mutant p53 proteins. A recent study highlighted the role of autophagy in the effects of the Hsp90 inhibitor, 17-AAG, in the elimination of mutant p53 protein in AML cells [123]. The p53 mutant harboring a host spot mutation at codon 248 (R248Q) with gain-of-function activity was reported to show an invasive activity in lung cancer cells [124] and a loss of tumor suppressor function for the oncogenic activity of the WT1 gene in AML [125]. Interestingly, the mutant p53^R248Q^ in AML cells is degraded by macroautophagy triggered by an Hsp90 inhibitor (17-AAG). Also, under metabolic stress conditions, 17-AAG triggers interaction between p53^R248Q^ and Hsc70, leading to chaperone-mediated autophagy to degrade p53^R248Q^ [123]. These data can facilitate future studies that could clarify the functional involvement of several types of autophagy and decipher the molecular mechanisms to improve anticancer therapies against AML harboring various *TP53* variants. A greater understanding of the distinct roles of macro-, micro-, chaperone-mediated, and other selective types of autophagy in removing mutant p53 proteins will promote the development of new therapeutic strategies for AML and other cancer cells.

#### *U2AF1* mutation, WWP1, and autophagy

*U2AF1* mutation at S34F promotes autophagic flux by activating FOXO3a, thereby mediating myelodysplastic syndrome progression [126]. Expression of the WW domain-containing E3 ubiquitin ligase 1 (WWP1), an oncogenic factor, is significantly increased in patients with AML and in AML cell lines [127]. The depletion of WWP1 in AML suppresses leukemic cell survival through activating autophagic signaling [127], suggesting WWP1 as a potential biomarker and therapeutic target for AML.

#### Epigenetic alterations of AML in the context of autophagy

The recent technological advances led us to identify several mutations of the epigenetically relevant genes, including *IDH1/2*, Tet methylcytosine dioxygenase 2 (*TET2*), DNA methyltransferase 3A (*DNMT3A*), and additional sex combs-like (*ASXL*)*1*, all of which are associated with the pathogenesis of AML [128–130]. Despite this, few studies show how dysregulation of autophagy is linked to the pathogenesis of epigenetically altered AML. Recurrent mutations of *IDH1* or *IDH2* in AML are strongly associated with DNA hypermethylation, and *DNMT3A* is responsible for *IDH*^mut^-associated hypermethylation [128]. In addition, the mutations of epigenetic modifier *TET2* are linked to the hypermethylation of the gene enhancer regions involved in leukocyte differentiation in hematopoietic stem cells, often observed in the premalignant status [129]. As a tumor suppressor, the global autophagy regulator TFEB is required to regulate *IDH1/2* expression and induces myeloid differentiation by controlling IDH1/2-TET axis [111]. However, the exact mechanisms by which TFEB-induced changes in genetic landscape affect the IDH1/2-TET2-mediated epigenetic changes are yet to be discovered. These efforts may facilitate the development of targeting autophagy in combination with DNA hypomethylating to treat AMLs harboring certain types of epigenetic alterations.

The germline duplication of *ATG2B* and *GSKIP* is related to a familial predisposition to myeloid malignancies and partly associated with *TET2*, but not *DNMT3A*, mutation [131, 132]. Although *Atg2b* and *Gskip* are required to maintain hematopoietic stem cell numbers and hematopoiesis [133], the autophagy regulation of these genes in AML tumorigenesis is unclear. A further complication in the genetic mutations of AML in studying the relevance of autophagy contribution is because the fusion oncogene AML1-ETO may collaborate with the mutations of *ASXL1/2* for AML tumorigenesis [134]. Given the discussed findings that ULK1-induced autophagy suppresses AML1-ETO9a-driven tumorigenesis of AML [91], it would be interesting to study how autophagy/ATGs are involved in the leukemogenesis in the combined mutations/fusion oncogene-linked AML.

Collectively, these data suggest the complicated involvement of autophagy and ATGs depending on the genetic alterations in AML. Additional studies are needed to further characterize the functional significance of autophagy in various genetic mutations in AML cells and clarify the molecular mechanisms by which distinct types of AML mutations regulate autophagic features for inducing leukemogenesis or anticancer effects.

### Function and regulation of ATGs in AML

#### ATGs as biomarkers of AML

Decreased Beclin-1 and p62 levels are associated with unfavorable outcomes of AML [135], suggesting the potential of autophagy genes as biomarkers for hematologic tumors. Although the evidence is inadequate, studies have supported the mediation of AML tumorigenesis by the bioinformatic tools through prediction of risk scoring based on autophagy gene signatures [136, 137]. In addition, profiling of genome-wide alternative splicing (AS) events in the AML cohort of The Cancer Genome Atlas indicates that survival-related AS events are enriched in leukemia-associated gene sets, including the autophagy pathway [138, 139]. Given that AS variants affect the structures and protein–protein interactions of proteins implicated in carcinogenesis [140], autophagy-related genes with aberrant AS events might be involved in AML pathogenesis.

Indeed, growing efforts have been made to identify specific ATGs as emerging biomarkers to predict clinical outcomes in AML. Recent studies showed that AML progression is dependent on the compositional signature of autophagy genes. Univariate Cox regression analysis and least absolute shrinkage and selection operator (LASSO) regression identified a six-gene signature (*CASP3, CHAF1B, KLHL24, OPTN, VEGFA,* and *VPS37C*) predictive of AML progression and survival [137]. A recent bioinformatics study using 10 ATGs for prediction of AML prognosis showed that groups at high risk of AML have higher expression of immune checkpoint genes and a greater proportion of CD4 T and NK cells [136]. According to univariate Cox regression analysis of the transcriptomic profiles of patients with AML, 32 ATGs were significantly associated with OS. LASSO Cox regression identified a critical risk signature for AML (*BAG3, CALCOCO2, CAMKK2, CANX, DAPK1, P4HB, TSC2,* and *ULK1*), which had excellent predictive power for AML prognosis [141]. Interestingly, patients at high risk of AML, as predicted based on an autophagy-related signature, exhibited a significantly increased immunosuppressive tumor microenvironment [141]. Moreover, the expression of WD-repeat protein interacting with phosphoinositides-1 (WIPI-1), the PI3P effector, was significantly decreased in samples from patients with AML (complex karyotypes; t(8;21); t(15,17); inv(16)) [142]. However, the effectiveness of ATGs currently remains an open question in the context of the translation of the ATG-related prognostic approaches to the clinic. Thus, the clinical utility of bioinformatics-based ATG signatures and the molecular mechanisms for autophagy-dependent immune regulation in AML pathogenesis warrant further investigation.

#### Genetic polymorphisms of ATGs in AML pathogenesis

Patients carrying the ATG10rs1864182G allele have a decreased risk of AML (odds ratio [OR] = 0.58, *p* = 0.001). In contrast, those homozygous for the ATG10rs3734114C allele have an increased risk of AML (OR = 2.70, *p* = 0.004). A functional analysis of *ATG10* genetic polymorphisms showed that the ATG10rs1864182G allele is associated with decreased autophagic flux, LC3-II degradation, and p62 accumulation compared with homozygous major allele carriers [57]. However, it is unclear how ATG polymorphisms affect autophagy in AML cells to influence AML tumorigenesis. Further studies are needed to predict the protein structural changes caused by single nucleotide polymorphisms in ATGs and the biological implications thereof. It is possible that several ATG variants have non-canonical autophagy-independent functions [143], which needs to be examined in the context of AML tumorigenesis.

#### Functions of ATGs in the pathogenesis of AML

ATG5 expression is elevated in mesenchymal stem cells from patients with AML, and silencing of ATG5 enhances the chemosensitivity of AML cells to genotoxic agents [144]. In addition, some immune factors that regulate autophagy play a critical role in leukemic responses in AML. CXCR4 expression is an independent prognostic factor for survival of patients with AML, and activation of CXCR4 signaling upregulates autophagic activity in AML cells [145]. SDF-1α/CXCR4 signaling interacts with crucial autophagy proteins, such as ATG5 and LC3, to improve AML survival. These data suggest that targeting SDF-1α/CXCR4/autophagy signaling may enhance therapeutic efficacy by targeting key ATGs [145].

#### Epigenetic regulation of ATGs in AML pathogenesis

A number of miRNAs have been implicated in AML tumorigenesis and chemoresistance and may acts as biomarkers [146]. For example*,* miR-17-5p, the expression of which is increased in leukemia [147, 148], promotes AML proliferation by inhibiting autophagy by targeting *BECN1* [149]. Ganesan et al. showed that stromal cells downregulate miR-23a-5p in leukemic cells to upregulate protective autophagy, thereby enhancing resistance to chemotherapy toxicity [150]. In addition, miR-143 sensitizes AML cells to Ara-C-induced cytotoxicity by inhibiting autophagy through targeting ATG7 and ATG2B [151]. Interestingly, co-expression of *ATG7* and *ATG2B* attenuated the effect of miR-143 on mitochondrial- and caspase-dependent apoptosis in Ara-C-treated AML cells [151]. miR-15a-5p, which is overexpressed in chemoresistant AML patients [152], has been discovered to target ATGs (*ATG9A, ATG14, GABARAPL1,* and *SMPD1*) to induce chemoresistance in AML cells [153]. Further investigations are needed to identify how miRNAs targeting specific ATGs are connected to other biological pathways such as caspase-dependent apoptosis in AML cells.

**Fig. 3:**
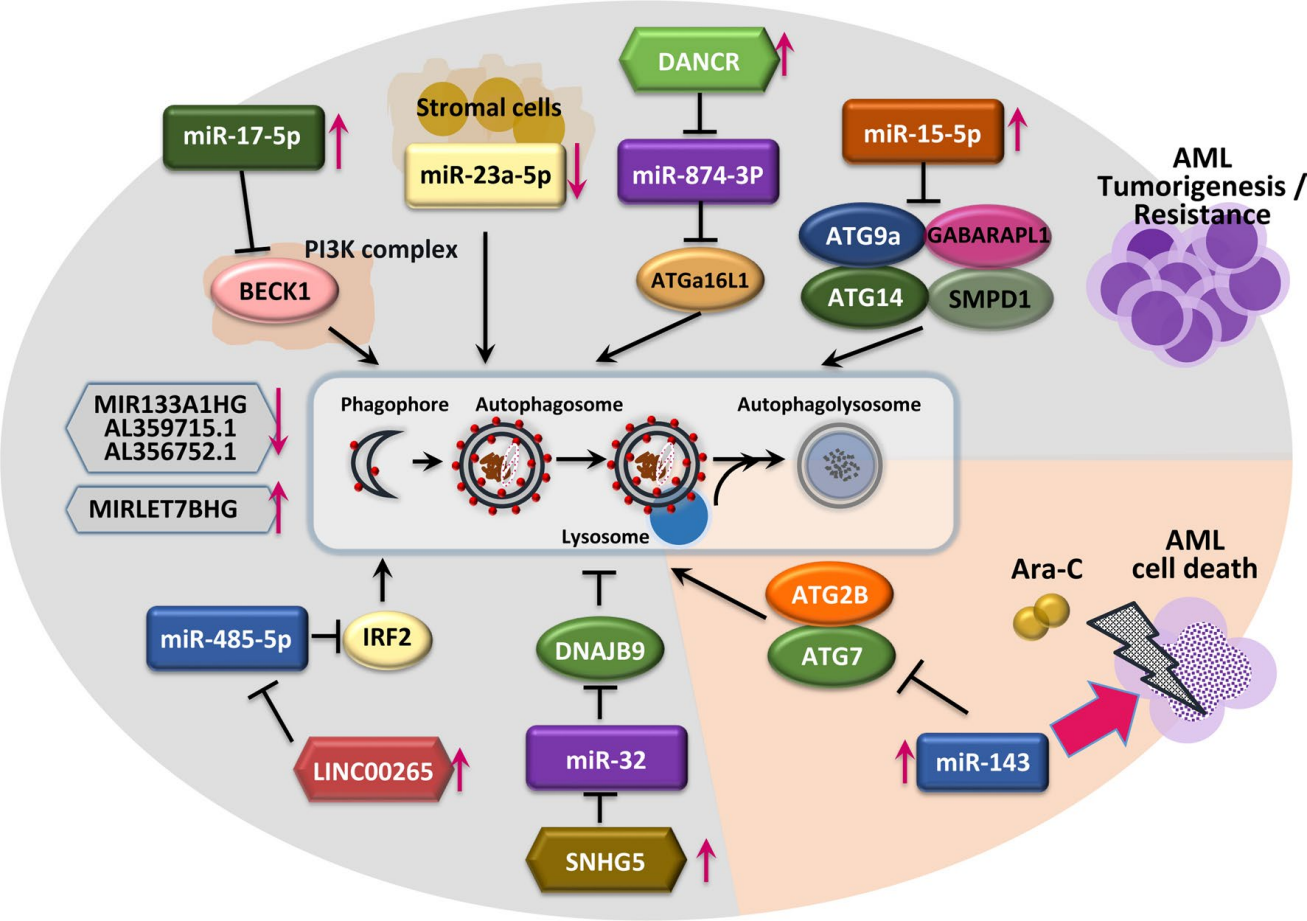
Epigenetic regulation of ATGs in AML. Various miRNAs, including miR-17-5p, miR-23-5p, miR-20-5p, miR-15-5p, and miR-485-5p, are related to AML tumorigenesis or chemoresistance by modulating autophagy via the regulation of various targets. Ara-C-induced differentiation antagonizing non-protein coding RNA (DANCR) promotes protective autophagy via the targeting of *miR-874-3p* to further induce ATG16L1 protein expression. LINC00265 acts as an endogenous competitive RNA for miR-485-5p, enhancing expression of IRF2 and attenuating apoptosis through autophagy activation. By contrast, miR-143 suppresses the *ATG7* and *ATG2B* genes, thereby increasing the cytotoxicity of Ara-C. Aberrantly expressed *SNHG5* gene in AML downregulates miR-32 and increases the DNAJB9 protein, the target of miR-32, acquiring chemoresistance through autophagy regulation. Autophagy-related long non-coding RNAs (MIR133A1HG, AL359715.1, AL356752.1, and MIRLET7BHG) are associated with poor prognosis in AML patients

Several lncRNAs have been described as oncogenes or tumor suppressors [154], and they play crucial roles in AML tumorigenesis. The lncRNA differentiation antagonizing non-protein coding RNA enhances Ara-C resistance in AML. Mechanistically, autophagy is activated in Ara-C-treated AML cells by suppressing miR-874-3p expression via enhanced expression of *ATG16L1*, a critical factor of autophagy [155]. Zhang et al. reported that the lncRNA LINC00265 promotes autophagy to attenuate AML cell apoptosis by competitively binding miR-485-5p to regulate IRF2 expression [156]. A genome-wide lncRNA expression study of pediatric patients with AML showed that small nucleolar RNA host gene 5 (*SNHG5*) is overexpressed in AML, and its silencing increased the chemosensitivity of AML cells by autophagy regulation via the miR32/DNAJB9 axis [157]. Although the role of DNAJB9 in regulating autophagy to influence chemotherapy resistance is unknown, the SNHG5/miR-32/DNAJB9 axis could be an efficient target for developing modalities to increase chemosensitivity in AML patients. Also, a recent study found that a signature containing four autophagy-related lncRNAs (MIR133A1HG, AL359715.1, MIRLET7BHG, and AL356752.1) has the potential as a biomarker to predict the survival of patients with AML [158]. Moreover, a preprint version of a study suggested that an autophagy-related lncRNA signature containing six lncRNAs (HYMAI, MIR155HG, MGC12916, DIRC3, C1orf220, and HCP5) has important prognostic value [159]. The epigenetic controls of ATG levels and their consequences of AML tumorigenesis and cell death are illustrated in Fig. 3. These findings suggest the functions of autophagy-related small RNAs to be context-dependent in AML, i.e., leukemogenesis, chemoresistance, or anticancer effects. Thus, the targeted approaches that boost or modulate ATG functions to develop novel therapeutics for AML patients, in particular, chemoresistant AML would be warranted.

### Targeting autophagy affecting tumor metabolism in AML pathogenesis

A growing body of evidence suggests that tumor cell metabolism is critically essential for the tumor cell survival, progression, metastasis, and development of resistance to various anticancer therapies [160–163]. Much effort has been devoted to understanding the metabolic status of numerous types of cancers in the context of autophagy regulation. In this section, we describe the current understanding of the crosstalk between autophagy and metabolism and its impact on identifying alternative therapeutic opportunities for AML.

#### Autophagy-mediated regulation of aerobic glycolysis in AML cells

Several cell lines exhibit differential metabolic profiles during AML pathogenesis [164–166]. KG-1 cells upregulate mitochondrial oxidative phosphorylation metabolism, which is associated with increased autophagy, whereas NB-4 and HL-60 cells have a glycolytic profile with low autophagic flux [164]. Pharmacological inhibition of the mTOR pathway upregulates autophagy and inhibits the glycolysis of polyploidy AML cells [167], suggesting a potential combination treatment for AML. However, human AML blasts show decreased expression of autophagy genes and autophagic flux with the accumulation of unhealthy mitochondria, contributing to tumor growth in AML [75]. Whether individual patients have distinct autophagic and metabolic profiles in AML cells should be evaluated to promote the development of personalized chemotherapeutics.

Although the mechanisms are unclear, syrosingopine, an inhibitor of the monocarboxylate transporter MCT4 and an autophagy inducer, has anti-proliferative activity in AML and enhances leukemic cell sensitivity to chemotherapeutic drugs [168]. MCT4 removes excess lactate, and future studies should assess the role of syrosingopine in the link between autophagy and lactate metabolism regulation in the context of cancer cell death. Interestingly, l-asparaginase inhibits mTORC1 activity, inducing strong apoptotic and autophagic responses in AML cells [169]. These data suggest that targeting glutamine metabolism is a promising therapeutic strategy for AML, although the mechanism is unclear. Inhibitors of the mTORC1/S6K1 pathway yielded mixed findings in a clinical study [170–172]. Therefore, further preclinical and clinical studies are needed to assess the utility of autophagy-targeting and glycolysis-modulating strategies in AML.

#### Autophagy crosstalk with oxidative phosphorylation and lipid metabolism

Mitochondrial oxidative phosphorylation (OXPHOS) activates autophagy and is implicated in the tumorigenesis, drug resistance, and relapse of solid and blood cancers [173, 174]. The autophagy pathway plays a role in lipid catabolism, providing free fatty acids to support OXPHOS in AML blasts, but not in normal hematopoietic cells. Indeed, autophagy inhibition in AML cells leads to accumulation of lipid droplets, thereby suppressing OXPHOS and inhibiting tumor cell proliferation and growth [173]. Further studies are needed to assess crosstalk between autophagy and mitochondrial function in AML blasts, which typically have an exacerbated metabolic profile compared with normal cells [175, 176]. Autophagy contributes to AML tumorigenesis by maintaining fatty acid oxidation via activation of lipophagy, thereby supporting mitochondrial OXPHOS [8]. Indeed, mitochondrial OXPHOS activation is a crucial distinguishing feature of chemotherapy-resistant cells [177].

**Fig. 4:**
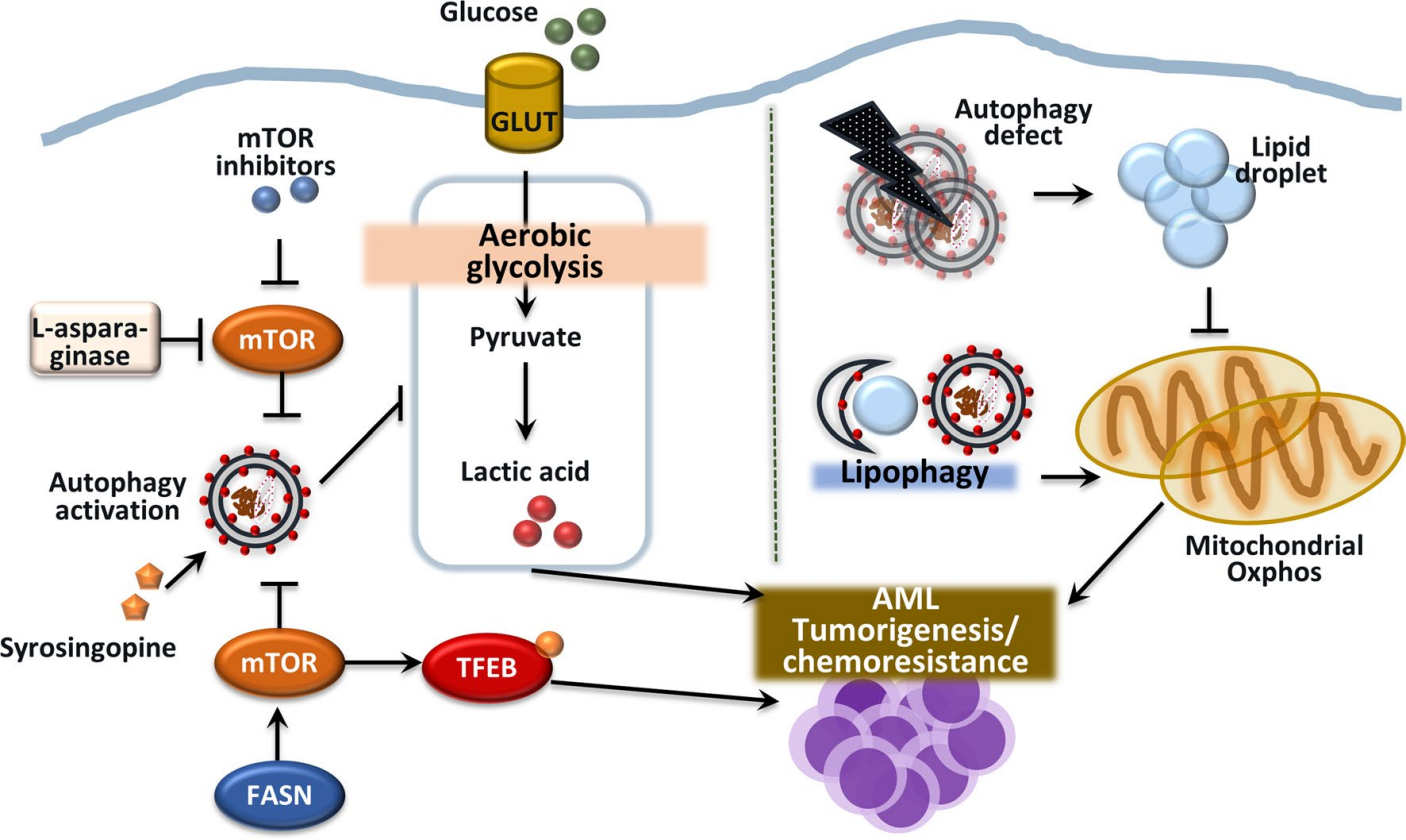
Autophagy crosstalks with tumor metabolism in AML pathogenesis. Distorted autophagy in the heterozygous loss of Atg5 protein in AML alters cell metabolism to aerobic glycolysis and enhances its aggressiveness. Decreased glycolysis and accumulation of lactate by syrosingopine (MCT4 inhibitor) inhibits cell proliferation through autophagic cell death. Glutamine depletion through L-asparaginase inhibits the mTOR pathway, strongly activating autophagy and apoptosis. AML with overexpressed *FASN* gene enhances mTOR signaling to sequester the transcription factor TFEB in the cytoplasm, thus blocking activities of leukemic cell differentiation. Lipid metabolism through autophagy is crucial to maintain the OXPHOS function in AML. When lipophagy is blocked, lipid droplets accumulate in AML cells, which suppress mitochondrial OXPHOS and cause cell death

Fatty acid synthase (FASN), a lipogenic enzyme for de novo fatty acid synthesis, promotes leukemic tumorigenesis by activating the mTOR pathway, thereby sequestering TFEB in the cytoplasm. The *FASN* mRNA level is significantly increased in AML blasts compared with healthy granulocytes or CD34 + hematopoietic progenitors. Inhibition of FASN expression in combination with ATRA treatment promotes myeloid-leukemic differentiation, at least in part by increasing TFEB activation and lysosomal biogenesis [178]. Therefore, promoting FASN degradation by activating autophagy may have therapeutic potential for AML. The interconnection between autophagy and tumor cell metabolism is shown in Fig. 4. Further studies are needed to evaluate the molecular mechanisms underlying the interplay between mitochondrial metabolism and autophagy in the pathogenesis of AML.

### Autophagy-modulating strategies for treatment of AML

A greater understanding of the mechanisms underlying the dual functions of autophagy will promote the development of novel therapeutic modalities. This section describes the previous and more recent works of literature that presented anti-leukemic effects of small molecules and agents through activation or inhibition of autophagy and a dual activation of apoptosis and autophagy.

#### Activation of autophagy

Targeting autophagy pathways in AML is challenging. There are several approaches to enhance autophagy to overcome AML chemoresistance. Because the mTORC1/S6K1 pathway is critical for the regulation of autophagy in AML initiation and progression, mTORC1/S6K1 inhibitors, such as RapaLink-1, show therapeutic potential for drug-resistant AML [170]. In addition, vitamin D, which inhibits the mTOR pathway, induced differentiation, inhibited growth, and induced Go/G1 arrest in several AML cell types [179–181]. Decreased levels of 25(OH)D3 and Beclin-1 are related to a poor prognosis for AML patients [149]. Given that vitamin D activates autophagy in hematopoietic cells and cancer cells [182, 183], it would be interesting to determine whether its therapeutic potential involves autophagy.

Considering the potential for therapeutic autophagy, several small molecules and agents have been reported to show a therapeutic benefit in AML cells and preclinical models of AML. For example, dendrogenin A (DDA), a mammalian cholesterol metabolite, exerts strong anti-leukemic effects in AML cell lines and primary AML tumors by activating autophagic cell death and synergistically enhancing the cytotoxic effects of Ara-C in AML cells and in vivo [184, 185]. The activity of DDA in inducing lethal autophagy contributes to its anti-leukemic effects on AML cells in vitro and in vivo. DDA, as a partial agonist for liver-X-receptor triggers Nur77- and NOR1-dependent autophagy, which is associated with increased LC3-II expression in AML cells [186]. Dihydroartemisinin, a drug used for malarial infection, inhibits AML cell growth by inducing ferroptosis of AML cells in an iron-dependent manner. Mechanistically, dihydroartemisinin-mediated autophagy via regulating AMPK/mTOR/p70S6k signaling is critical in the degradation of ferritin, accumulation of reactive oxygen species, and ferroptotic cell death [187]. A recent study also showed that the anti-leukemic effect of the small molecule AC-73, alone or in combination with Ara-C or ATO, is mediated at least in part by autophagy activation [188].

The myeloid differentiation effect of ATRA is accompanied by autophagy induction in AML cells [189, 190], although the mechanism is unclear. In addition, the therapeutic effect of ATRA on ATRA-sensitive AML and APL cells depends on autophagy-linked FYVE domain protein, which is critical for aggrephagy induction and ATRA-induced proteolysis [77]. In addition, tumor protein p73-mediated death-associated protein kinase 2 (DAPK2) signaling mediates ATRA differentiation therapeutics via autophagy activation in APL cells [191]. The DAPK2–ATG5 interaction is required for ATRA-induced autophagy in APL cells [191]. Therefore, autophagy-activating agents have therapeutic promise for AML, although these have not been translated effectively within a clinical setting. Therefore, future clinical trials are needed to clarify the efficacy of autophagy-activating agents combined with conventional drugs against AML.

#### Inhibition of autophagy

##### Autophagy pathway inhibitors

Cytoprotective autophagy has been implicated in the initiation and maintenance of myeloid leukemia [70]. Autophagy inhibitors have therapeutic potential for AML. The cytotoxic effects of the autophagy blockers hydroxychloroquine and/or bafilomycin A1 (BafA1) are more prominent in Ara-C-resistant U937 cells [192] and KG-1 cells than in HL-60 cells when exposed to ATO and ATRA [193]. Moreover, the in vivo therapeutic effects of BafA1 with Ara-C support their potential as novel therapeutics for AML [194]. In addition, leukemia stem cells (LSCs) showing resistance to bromodomain and extraterminal domain inhibitors exhibited upregulation of BECN1/Beclin-1 and increased autophagy via activation of the AMPK (p-Thr172)/ULK1 (p-Ser555) pathway. Blockade of AMPK pathway activation increased apoptotic death induced by a bromodomain and extraterminal domain inhibitor in AML LSCs [195]. However, autophagy inhibition does not affect Kmt2a/Mll-Mllt3/Af9 AML cells, which have marked autophagic flux. Chloroquine is ineffective in vivo and induces drug resistance in AML cells by vesicular exocytosis [196]. Therefore, the effects of macroautophagy inhibitors warrant further investigation in preclinical and clinical studies.

The autophagy inhibitor hydroxychloroquine has a cytotoxic effect, particularly on CD34-expressing/low-ROS-generating AML cells with high basal autophagy [119]. Indeed, a subgroup of patients with AML exhibits increased susceptibility to chloroquine [197]. These patients show upregulation of 99 genes, most of them related to leukemogenesis [197]. Although chloroquine exerts an anti-leukemic effect, patient heterogeneity should be considered [197]. In addition, autophagy inhibitors do not re-sensitize Ara-C-resistant AML cells to Ara-C [198]. Thus, autophagy-targeting therapy is effective only for a particular group of patients, who should be selected by accurate risk scoring using improved bioinformatic tools. Another autophagy inhibitor, SAR405, is a highly potent small-molecule inhibitor of phosphatidylinositol 3-kinase, catalytic subunit type 3 (PIK3C3)/Vps34 and blocks autophagy to reduce AML cell proliferation and the repopulating capacity of *FLT3*-ITD AML cells [93, 199]. Considering the potent and selective effects of SAR405 on the late endosome–lysosome and autophagy pathways [200, 201], it would be interesting to explore the anti-leukemic activity of SAR405 in detail in future.

Combination therapeutics may exert a synergistic effect. For example, the combination of an autophagy inhibitor (chloroquine and BafA1) and a histone deacetylase inhibitor enhanced AML cell death, particularly in AML expressing *AML1-ETO* [12]. In addition, the combination of an autophagy inhibitor (chloroquine or LY294002) and a CDK4/6 inhibitor synergistically induced apoptosis of AMLs [202]. Because CDK4/6 inhibitors suppress t(8;21) AML cell proliferation, enhance autophagosome formation, and induce autophagy [202], co-inhibitory strategies for CDK4/6 and the autophagy pathway may be effective for treating t(8;21) AML. More data are needed on the effects of autophagy inhibitory therapeutics on Ara-C efficacy against Ara-C-resistant AML.

##### Targeting ATGs or autophagy proteins

Silencing of ATG7 in AML cells drives proapoptotic phenotypes and enhances chemosensitivity to Ara-C and idarubicin [203, 204]. Atg7 inhibition is associated with decreased leukemia-initiating cells and prolonged survival of immunodeficient mice with disseminated AML [70, 203, 204], suggesting a therapeutic role for ATG7 inhibition in AML. Autophagy may initiate leukemogenesis in AML expressing the oncogene *MLL-AF9* because Atg5 deficiency led to malignant transformation and AML progression in a mouse model of *MLL-AF9*-driven AML [89]. Therefore, several ATGs may contribute to AML initiation and development. WAVE1, a WASP family member and a verprolin-homologous protein, promotes autophagy activation via Beclin-1/Bcl-2 and Beclin-1/PI3K-III complex-dependent pathways, thus augmenting the survival and chemoresistance of AML [205].

Transient receptor potential melastatin 2 (TRPM2), an ion channel highly expressed in AML, activates autophagy by regulating key transcription factors, ATF4 and CREB, in autophagosome biogenesis [206]. Silencing of TRPM2 reduced the ULK1, ATG7, and ATG5 protein levels and cancer cell proliferation and increased doxorubicin sensitivity in vitro and in vivo [206]. Thus, targeting TRPM2-mediated autophagy may have therapeutic potential and may increase chemosensitivity in AML. Another autophagy protein, vacuole membrane protein (VMP1), is increased in a subset of patients with AML and amplifies autophagic flux and lysosomal degradation. Although VMP1 overexpression increased autophagy and mitochondrial quality control, it protected against venetoclax-induced apoptotic cell death [207].

##### Blockade of selective autophagy

BafA1 suppresses in vivo maintenance of AML stem cells in a mouse model of AML [194]. The effects of autophagy inhibitors on leukemia stem cells and AML blasts are mediated by mitochondrial damage, mitophagy inhibition, and mitochondrial homeostasis disruption [194]. Two mitophagy genes, *BNIP3L* and *P62*/*SQSTM1*, are potential prognostic markers in AML [65, 66]. Interestingly, knockdown of BNIP3L/Nix or p62/SQSTM1 influenced mitochondrial function and increased sensitivity to mitochondria-targeting agents [65]. These findings suggest mitophagy receptors to be useful therapeutic targets in AML. Silencing of p62/SQSTM1 decreased leukemia cell growth, which was associated with defective mitophagy, *i*.*e*., delayed removal of dysfunctional mitochondria and impaired mitochondrial respiration [66]. The progenitor potential and maintenance of human AML LSCs is dependent on AMPK/FIS1-mediated mitophagy. FIS1 depletion leads to myeloid differentiation and induces cell cycle arrest in AML cells. Therefore, the components of mitochondrial dynamics may modulate stem cell properties in AML tumorigenesis [208]. These data suggest that selective autophagy activation contributes to leukemia progression by modulating mitochondrial quality control and respiration.

##### Small-molecule autophagy inhibitors

Several small molecules and chemicals reportedly enhance antitumor cytotoxic responses by inhibiting autophagy in AML cells. THZ-P1-2 targets unique cysteines within PI5P4K, a kinase involved in tumorigenesis, and exerts an anticancer effect on leukemia cells by impairing autophagic flux and inducing lysosomal dysfunction [209]. The antitumor activity of VPS34-IN1, a specific inhibitor of VPS34 [210], is mediated by suppression of L-asparaginase-induced autophagy and impairment of mTORC1 signaling in AML cells [211]. XRK3F2, a specific inhibitor of the selective autophagy receptor p62/SQSTM1, promotes death of leukemia-initiating cells in AML. The effect of XRK3F2 is mediated by inhibition of mitophagy via blockade of the p62 interaction with damaged mitochondria, thereby impairing AML progression [212]. Several epigenetic inhibitors, including histone deacetylase inhibitors, exert an anti-leukemic effect at least in part by modulating the autophagy pathway [213]. Chidamide, a novel benzamide histone deacetylase inhibitor, enhances the AML cell cytotoxicity of the chemotherapy drugs Ara-C and sorafenib by upregulating histone H3 lysine 9 trimethylation (H3K9me3) and downregulating autophagy by inhibiting SIRT1 expression [214]. In screening for a small molecule that exerts a synergistic effect with AC220 (quizartinib), which is an FLT3 receptor tyrosine kinase inhibitor, TAK-165, which is an HER2 inhibitor, induced death of various types of cancer cells. Although TAK-165 inhibits autophagy in an HER2-independent manner, the anticancer effect of TAK-165 and AC220 is dependent on activation of chaperone-mediated autophagy [215].

Much effort has focused on designing agents that bind with high affinity to the LIR motifs of selective autophagy receptors. Agents with high-affinity binding to the LIR motifs of three selective cargo receptors (OPTN, p62, and NDP52) increase AML cell sensitivity to Ara-C [216]. We anticipate the development of efficient small-molecule inhibitors of selective autophagy that increase the sensitivity of AML blasts to chemotherapy. Tables 1 and 2 summarize the autophagy-modulating small molecules and/or agents used in the development of AML treatment.

**Table 1:** Small molecules and/or agents for activation of autophagy in the context of AML treatment

Agents	Known for	Mechanism	Outcome/effects	Study model	Ref
Vitamin D		Inhibition of miR-17-5p and induction of Beclin-1,	Inhibition of cell proliferation	HL-60, AML patients sample	[149]
Dendrogenin A	A mammalian cholesterol metabolite	LXRβ-dependent sensitization of AML cells to Ara-C in vitro and in vivo	Potentiation of Ara-C cytotoxicity	HL-60, KG1, MV4-11, AML patients samples, AML xenograft in mice	[185]
		Inhibition of phosphorylation of Akt and JNK to maximize the idarubicin induced DNA damage and lethal autophagy	Potentiation of Idarubicin-induced cell death	KG1α, KG1, MOLM14, OCI-AML3, AML patients samples, xenograft in mice	[184]
		LXRβ-, Nur77-, and NOR1-dependent induction of lethal autophagy	Anti-leukemic effect	KG1, HL-60, AML patients samples, AML xenograft in mice	[186]
Dihydroartemisinin	Anti-malarial drug	Inhibition of mTOR/p70S6k signaling and activation of AMPK leading to autophagy dependent ferroptosis	Induction of ferroptosis, inhibition of cell/xenograft growth	HL-60, KG1, THP-1, AML xenograft in mice	[187]
AC-73	Specific inhibitor of CD147	Inhibition of ERK/STAT3 signaling and potentiation of ATO-induced autophagy	Inhibition of cell proliferation	U937, NB4, HL-60, MV4-11, AML patients samples	[188]

*LXRβ* oxysterols receptor LXR-beta, *Ara-C* cytarabine, *JNK* c-Jun N-terminal kinase, *Nur77* transcription factors NR4A1, *NOR1* transcription factors NR4A3, *CD147* cluster of differentiation 147, *ATO* arsenic trioxide

**Table 2:** Small molecules and/or agents for inhibition of autophagy in the context of AML treatment

Agents	Known for	Mechanism	Outcome/effects	Study model	Ref
Bafilomycin A1	Inhibitor of vacuolar H^+^-ATPase	Inhibition of autophagy accumulates the damaged mitochondria	Effective cytotoxicity in hypoxic condition	HL-60, MOLM-13, AML patients samples	[194]
Hydroxy-chloroquine	Anti-malarial drug	Inhibition of autophagy activates caspase 9 in Ara-C-resistant AML cells	Inducing intrinsic mitochondria apoptosis	U937, OCI-AMLM-2, AML patients samples	[192]
		Inhibition of autophagy converted CD34( +)/ROS^low^ AML cells to CD34( +)/ROS^high^ AML cells	Increasing ROS production and inducing apoptosis	HL-60, K562, THP1, OCIM3, MOLM13, NB4, AML patients samples	[119]
SAR405	VPS34 inhibitor	SAR405 inhibits the autophagy process in *FLT3*-ITD AML cells	Inhibition of proliferation and Inducing apoptosis	MV4-11, MOLM-14, OCI-AML3, AML patients samples	[199]
			Impairment of proliferation	MV4-11, MOLM-14, OCI-AML3, AML patients samples	[93]
THZ-P1-2	PI5P4K inhibitor	Lysosomal–autophagosomal defect and increased TFEB activation (mechanism studied in HeLa cells)	Anti-proliferative	THP1, OCI/AML-2, SKM1	[209]
VPS34-IN1	VPS34 inhibitor	Reduction of intracellular vesicle trafficking, inhibition of basal and L-asparaginase-induced autophagy, modulation of mTORC1 and FLT3-ITD signaling	Mitochondrial apoptotic cell death/Anti-leukemic	HL-60, MOLM-14, several AML cell lines	[211]
XRK3F2	Inhibitor of ZZ domain of p62	Inhibition the binding of p62 with defective mitochondria to block mitophagy	Inhibition of leukemia-initiating potential of leukemia cells	K562, HL-60, patient-derived tumor xenograft model	[212]
Chidamide	HDAC inhibitor	Inhibition of SIRT1 expression to inhibit the Ara-C or sorafenib-induced autophagy	Enhancement of cytotoxicity of chemotherapy drugs	THP-1, MV4-11	[214]
TAK-165	HER2 inhibitor	HER2-independet inhibition of autophagy, but induction of chaperone-mediated autophagy (CMA) during TAK-165/AC220 combinatorial treatment	Enhanced efficacy of AC220 to induce cancer cell death	HEL, ES-2, OCI-AML3	[215]

*Ara-C* cytarabine, *CD34* cluster of differentiation 34, *ROS* reactive oxygen species, *VPS34* vacuolar protein sorting 34, *FLT3-ITD* fms-like tyrosine kinase 3-Internal tandem duplications, *PI4P4K* phosphatidylinositol 5-phosphate 4-kinases, *TFEB* transcriptional factor EB, *HDAC* histone deacetylases, *HER2* human epidermal growth factor receptor 2, *AC220* FLT3 receptor tyrosine kinase inhibitor

#### Dual activation of apoptosis and autophagy

Induction of autophagy-mediated cell death when apoptotic death is bypassed is critical for killing refractory cancer cells. Indeed, several agents that induce apoptosis and autophagy have therapeutic potential for AML. For example, 4-amino-2-trifluoromethyl-phenyl retinate, an ATRA derivative, exerts an anticancer effect on AML cells by inducing ferroptosis via activation of the ROS/autophagy pathway [217]. A polysaccharide from eggs of *Strongylocentrotus nudus* activates both autophagy and apoptosis in AML cells, the latter effect being dependent on activation of the nuclear factor kappa-B signaling pathway [218]. However, how *S. nudus* activates autophagy to promote cytotoxicity in AML cells is unclear.

Numerous small molecules and drugs have been reported to activate both autophagy and apoptosis in the context of anti-AML responses. A dual inhibitor of mTORC1 and mTORC2 (AZD8055) showed an antitumor effect in AML through activation of autophagy and caspase-dependent apoptosis [219]. Metformin, an antidiabetic drug, induces an anti-leukemic effect by activating apoptosis and autophagy in *FLT3*-ITD AML [220]. Decitabine, a DNA methyltransferase inhibitor used for the treatment of AML, significantly inhibits the expression of *TP53*-induced glycolysis and apoptosis regulator (TIGAR), inducing an anti-leukemic effect through induction of apoptosis and activation of autophagy via upregulation of several ATGs [221]. In addition, the anti-leukemic effect of bortezomib (Velcade), a novel proteasome inhibitor, depends on TRAF6 degradation in myelodysplastic syndrome and AML cell lines and primary cells through a dual activation of the autophagy-mediated lysosomal pathway and apoptotic function [222]. Another study showed that the increased sensitivity of *FLT3*-ITD AML cells to bortezomib is related to both autophagy and apoptosis [223]. Interestingly, blockade of bortezomib-induced autophagy promotes apoptosis of NB4 cells [224]. Moreover, tanshinone IIA, a lipophilic active constituent, shows anticancer activity against AML cells by inducing autophagy and apoptosis via inhibition of the PI3K/Akt pathway, although blockade of tanshinone IIA-mediated cytoprotective autophagy enhances apoptotic cell death [225]. These data suggest that the dual mediators induce anticancer effects depending on the crosstalks between autophagy and apoptosis.

Du et al. showed that chlorprothixene, a dopamine receptor antagonist, suppresses the growth of AML cells by modulating multiple biological pathways and inducing apoptosis and autophagic cell death [226]. Sertraline, a widely used antidepressant, suppresses AML cell lines and primary AML cells by activating apoptosis- and autophagy-mediated cell death [227]. Matrine, an alkaloid from *Sophora flavescens* Ait, exerts an antitumor effect against AML by activating autophagy and apoptosis [228]. Indeed, an increased population of apoptotic and autophagic blast cells during the initial phase (12–24 h) after chemotherapy is related to a better outcome of AML [229], highlighting the predictive value of autophagy and apoptosis. A recent study reported that acetylshikonin (ASK), a natural naphthoquinone derivative of the Chinese medicine *Lithospermum erythrorhyzon*, induces both apoptosis and autophagy in AML cells. Interestingly, ASK has an effect of inhibiting cell viability and proliferation and promotes cell cycle arrest of AML cells. Moreover, ASK induces autophagic flux through enhancing AMPK and phospho-liver kinase B1 (LKB1) expression while inhibiting PI3K/Akt-mediated mTOR signaling pathways [230].

In silico screening identified a group of antihistamines that possess anti-leukemic effects against primary AML cells ex vivo [231]. The effects of antihistamines on AML are mediated by simultaneous disruption of mitochondrial and lysosomal function, suggesting dual targeting. In addition, antihistamines mediate cancer cell death by inducing autophagy and apoptosis [231]. Interestingly, the apoptotic enzyme caspase-3 modulates AML1-ETO9a-driven leukemogenesis by influencing ULK1-mediated autophagy in AML [91]. Therefore, caspase-3 modulates ULK1-mediated macroautophagy activation to limit AML1-ETO9a-driven leukemia. Further investigation of the mechanisms by which these agents induce apoptosis and autophagy in AML cells is needed because autophagy inhibits apoptotic cell death under certain conditions. Several agents, which have a dual mode of action in autophagy and apoptosis in the context of AML treatment, are summarized in Table 3. Where autophagy pathways or genes behave as targets for AML therapeutics, it will be challenging to find activators or inhibitors that specifically regulate the autophagy activity in LICs or leukemic cells with heterogeneous molecular properties in the current and future era of personalized medicine.

**Table 3:** Dual regulators of autophagy and apoptosis in AML treatment

Agent	Known for	Mechanism	Outcome/Effects	Study model	Ref
4-Amino-2-trifluoromethyl-phenyl retinate	ATRA derivative	Nrf2-mediated regulation of iron homeostasis to induce the autophagy dependent ferroptosis	Ferroptosis and promoting differentiation	HL-60, NB4, U937, AML xenograft in mice	[217]
SEP	Polysachharide	NF-κB-activated autophagy leading to apoptosis	Inhibition of leukemia progression	HL-60, L1210, murine AML allograft	[218]
Metformin	Anti-diabetic drug	Inhibition of mTOR signaling during metformin and sorafenib combined treatment	Synergistic inhibition of cell proliferation by metformin and sorafenib	MV4-11, primary FLT3-ITD mutated leukemia cells	[220]
Sertraline	Antidepressant drug	Induction of autophagy to facilitate apoptosis	Anti-proliferative effect and apoptosis	NB4, NB4-R1, NB4-R2, AML patients samples	[227]
Decitabine	DNA methyltransferase inhibitor	Downregulation of TIGAR to induce ROS-associated apoptosis, induction of autophagosomes and secondary lysosome formation	Autophagy and apoptosis	HL-60, KG1, MV4-11, AML patients samples, AML xenograft in mice	[221]
AZD8055	mTOR inhibitor	Inhibition of mTORC1 and mTORC2 signaling: inhibition of phosphorylation of eIF4E-binding protein and PI3K/Akt feedback activation	Autophagy and apoptosis	AML patients samples, MV4-11, AML xenograft in mice	[219]
Bortezomib	Proteasome inhibitor	Induction of ER stress, selective degradation of TRAF6 protein	Suppression of MDS/AML cell survival	TF-1, THP-1, HL-60, MDS-L, MDS/AML patients samples	[222]
		Autophagic degradation of FLT3-ITD protein through inhibition of MARK, PI3K/AKT, and STAT5 pathways	Activating lethal autophagy and apoptosis	OCI-AML3, MOLM-14, AML xenograft in mice	[223]
		Activation of autophagy prior to apoptosis	Autophagy and apoptosis	NB4, HL-60	[224]
Chlorprothixene	Dopamine receptor antagonist	Induction of auto-lysosome fusion and autophagolysosome formation, reduction of oncofusion protein PML-RARα and AML1-ETO	Apoptotic cell death and inhibition of AML tumor growth	NB3, Kasumi-1, K562, U937, AML xenograft in mice	[226]
Tanshinone IIA	Lipophilic active constituent from the root of *Salvia miltiorrhiza*	Inhibition of PI3K/Akt/mTOR pathway	Autophagy and apoptosis	U937, AML xenograft in mice	[225]
Acetylshikonin	Natural naphthoquinone derivative of *Lithospermum erythrorhyzon*	Activation of LKB1/AMPK pathways and inhibition of PI3K/AKT/mTOR pathways	Promoting autophagy-dependent apoptosis	HL-60, K562, THP-1	[230]
ANHA	Antihistamine	Increased ROS and reduced MMP, mitochondrial damage, and lysosomal disruption	Anti-leukemic in vitro, ex vivo, and in vivo	AML patients samples, several AML cell lines, AML xenograft in mice	[231]

*ATRA* all-trans retinoic acid, *SEP* *Strongylocentrotus nudus* egg polysaccharide, *Nrf2* nuclear factor erythroid 2-related factor 2, *mTOR* mammalian target of rapamycin, *TIGER* TP53-induced glycolysis and apoptosis regulator, *eIF4E* eukaryotic initiation factor 4E, *ER* endoplasmic reticulum, *TRAF6* TNF receptor-associated factor 6, *MDS* myelodysplastic syndrome, *ROS* reactive oxygen species, *PML-RARα* promyelocytic leukemia/retinoic acid receptor alpha, *LKB1* phospho-liver kinase B1, *ANHA* antihistamines

### Clinical studies of autophagy-targeting drugs in AML

Most clinical trials related to autophagy have evaluated mTOR1/2-related agents. In 2018, an mTORC1 inhibitor (sirolimus) and the mitoxantrone/etoposide/Ara-C regimen showed a higher overall response rate (ORR) in patients with than in those without baseline mTOR activation (71% vs. 20%) ([232], NCT00780104, NCT01184898). In the GOELAMS study, an mTOR inhibitor (RAD001, everolimus) plus Ara-C-based therapy improved the clinical outcomes of patients with AML. Surprisingly, patients with FLT3-ITD receiving RAD001 achieved a CR (75%, 3/4) ([233], NCT01074086). Some older AML patients who received everolimus with LDAC achieved an ORR (25%, 6/24) and partial remission (PR) (4.2%, 1/24) ([234], NCT00636922). An mTOR inhibitor combined with standard therapy improved the status of AML patients with a poor prognosis. Although an mTOR inhibitor plus conventional chemotherapy did not show a clinical benefit in two studies [235, 236], patients who responded to mTOR inhibitor (temsirolimus) by inhibiting phosphorylation of ribosomal protein S6 had a significantly higher CR rate (75% vs. 0%) [237]. Therefore, mTOR inhibitors are useful therapeutics for AML, and several clinical trials are underway (NCT00819546, NCT02638428, NCT01869114).

The ability of vitamin D to enhance the efficacy of conventional chemotherapy has been evaluated in clinical trials. In older patients with AML, calcitriol with LDAC and hydroxyurea resulted in a 79% ORR (CR 45%, PR 34%), confirming the therapeutic value of vitamin D [238]. Furthermore, the OS of older AML patients who did not respond to demethylating agents was longer after treatment with vitamin D and deferasirox than best supportive care (10.4 vs. 4 months) [239].

Velcade-induced autophagy modification may improve clinical outcomes. Some patients with relapsed AML who received velcade with idarubicin achieved a CR (20%) and most showed improvement of circulating blasts ([240], NCT00382954). The CALGB (Alliance) study 10,502 showed a higher remission rate in older patients with AML after conventional chemotherapy with velcade versus without velcade (65% in CALGB study 10,502 vs. 47% in CALGB study 9720) [241, 242]. Another clinical trial of velcade in older patients with AML is underway (NCT01420926). A trial of the autophagy inhibitor chidamide [214] demonstrated a clinical benefit in patients with relapsed/refractory AML, especially in those with epigenetic and transcription factor-related gene mutations (CR: 59.5% vs. 28.2% in patients with other mutations; *p* = 0.006)([243], NCT02886559). Table 4 summarizes the autophagy-modulating agents used in clinical trials for AML treatment.

**Table 4:** Autophagy-modulating agents used in AML clinical trials

Drug	Target mechanism	enrolled patients	Combination therapy	Phase (status)	Clinical outcomes (N)	Ref
Sirolimus	mTOR inhibitor (autophagy induction)	High-risk AML	Mitoxantrone, etoposide, and cytarabine (MEC)	Phase I&II (complete)	ORR: 47% ORR with baseline target inhibition: 71% ORR without target inhibition: 20%	NCT00780104 NCT01184898 [232]
RAD001 (everolimus)	mTOR inhibitor (autophagy induction)	First relapse AML (under 65 years)	7 + 3 (cytarabine + daunorubicin)	Phase Ib (complete)	CR: 68% (19/28) FLT3-ITD mutated patient achieved CR (3/4)	NCT01074086[233]
		Elderly AML	Low-dose Ara-C	Phase Ib (complete)	ORR: 25% (6/24) PR: 4.2% (1/24)	NCT00636922 [234]
Vitamin D	Autophagy induction	Elderly AML after treatment failure	Deferasirox	Retrospective case–control study	Median survival: 10.4 months vs 4 months (treated vs best supportive care)	[239]
Bortezomib	Autophagy induction	Elderly, eligible AML/relapsed AML	Weekly idarubicin	Phase I (complete)	CR: 20% (4/20) PR: 5% (1/20)	NCT00382954[240]
		Eligible AML	7 + 3 (cytarabine + daunorubicin)	Phase I&II (complete)	CR: 65% CRp: 4%	[241]
Chidamide	Autophagy inhibition	Relapse/refractory AML	Decitabine, cytarabine, aclarubicin, and granulocyte colony-stimulating factor	Phase I/II (complete)	CR: 25.8% (24/93) CRi: 20.4% (19/93) PR: 8.6% (8/93)	NCT02886559[243]

Further details for trial with NCT numbers can be accessed at http://clinicaltrials.go

*CR* complete remission, *CRi* complete remission with incomplete hematologic recovery, *CRp* complete remission with incomplete platelet recovery, *PR* partial response, *ORR* overall response rate

Although several clinical trials have yielded negative clinical results, autophagy modulators show therapeutic promise for AML, particularly for patients with a poor prognosis or who are ineligible for intensive chemotherapy. Sensitivity to autophagy-modulating agents depends on the patients’ characteristics and genetic mutations. Therefore, further studies are required to determine the role of autophagy-modifying agents in the treatment of AML.

## Conclusion

Despite investigations of targeted therapies for AML, outcomes remain poor in relapsed AML and older patients. Because dysregulation of autophagy contributes to genomic instability, autophagy suppresses the initiation of AML tumorigenesis. However, autophagy is essential for maintaining stem-like cancer cells and inducing chemoresistance during AML treatment. Therefore, autophagy seems to have both tumor-promoting and suppressing properties. The role of selective autophagy warrants further investigation. The molecular relationship between autophagy and oncogenic mutations in AML is only now beginning to be understood. The ATG signature has the potential to predict AML clinical outcomes. Because epigenetic regulation of autophagy is unclear, identification of the key regulators, *i*.*e*., miRNAs and lncRNAs, of autophagy in tumor cells will provide insight into the mechanisms of AML tumorigenesis.

Autophagy crosstalk with AML cell metabolism influences tumorigenesis and chemoresistance. However, how autophagy regulates the metabolic status of tumor cells and neighboring cells in the tumor microenvironment and the implications for personalized AML treatment are unclear. Investigation of agents and small molecules that activate or inhibit autophagy pathways has enhanced our understanding of autophagy-targeting therapeutics for AML. However, the clinical efficacies and the patients who should receive autophagy activators and inhibitors are unclear. A mechanistic understanding of the roles of autophagy will guide the development of autophagy-targeted personalized therapeutics for AML.

## Data Availability

Not applicable.

## Abbreviations

AML: Acute myeloid leukemia
AS: Alternative splicing
ASK: Acetylshikonin
AML-ETO: AML eight-twenty-one
AMPK: 5' Adenosine monophosphate-activated protein kinase
APL: Acute promyelocytic leukemia
Ara-C: Cytarabine
ASXL: Additional sex combs-like
ATF4: Activating transcription factor 4
ATG: Autophagy-related genes
ATO: Arsenic trioxide
ATP: Adenosine triphosphate
ATRA: All-trans retinoic acid
BafA1: Bafilomycin A1
CBF-AML: Core-binding factor AML
CD147: Cluster of differentiation 147
CR: Complete remission
CXCR4: C–X–C chemokine receptor type 4
DAPK2: Death-associated protein kinase 2
DDA: Dendrogenin A
DNMT3A: DNA methyltransferase 3A
DRAM2: DNA-damage-regulated autophagy modulator 2
eIF4E: Eukaryotic initiation factor 4E
ER: Endoplasmic reticulum
FASN: Fatty acid synthase
FLT3-ITD: Fms-like tyrosine kinase 3—internal tandem duplication
FOXO3: Forkhead box O3
HDAC: Histone deacetylases
HER2: Human epidermal growth factor receptor 2
HSC: Hematopoietic stem cell
JNK: C-Jun N-terminal Kinase
IDH: Isocitrate dehydrogenase
LASSO: Least absolute shrinkage and selection operator
lncRNA: Long non-coding RNA
LSCs: Leukemia stem cells
LICs: Leukemic-initiating cells
LKB1: Liver kinase B1
LXRβ: Oxysterols receptor LXR-beta;
MDS: Myelodysplastic syndrome
miRNA: MicroRNA
MLL: Mixed lineage leukemia
mTOR: Mammalian/mechanistic target of rapamycin
NOR1: Transcription factors NR4A3
Nrf2: Nuclear factor erythroid 2-related factor 2
Nur77: Transcription factors NR4A1
ORR: Overall response rate
OXPHOS: Oxidative phosphorylation
PIK3C3: Phosphatidylinositol 3-kinase, catalytic subunit type 3
PI4P4K: Phosphatidylinositol 5-phosphate 4-kinases
PKM2: Pyruvate kinase isoenzyme M2
PML/RARα: Promyelocytic leukemia/retinoic acid receptor alpha
RAS: Rat sarcoma virus
ROS: Reactive oxidative species
PR: Partial remission
SDF-1: Stromal cell-derived factor 1
SEP: *Strongylocentrotus nudus* egg polysaccharide
SNHG5: Small nucleolar RNA host gene 5
SQSTM1: Sequestosome 1
STAT3: Signal transducer and activator of transcription 3
TFEB: Transcriptional factor EB
TET2: Tet methylcytosine dioxygenase 2
TIGER: TP53-induced glycolysis and apoptosis regulator
TRAF6: TNF receptor-associated factor 6
TRPM2: Transient receptor potential melastatin 2
U2AF1: U2 small nuclear RNA auxiliary factor 1
ULK1: Unc-51-like kinase 1
VMP: Vacuole membrane protein
VPS34: Vacuolar protein sorting 34
WIPI-1: WD-repeat protein interacting with phosphoinositides-1
WWP1: WW domain-containing E3 ubiquitin ligase 1
